# Multitemporal Volume Registration for the Analysis of Rheumatoid Arthritis Evolution in the Wrist

**DOI:** 10.1155/2017/7232751

**Published:** 2017-10-19

**Authors:** Roberta Ferretti, Silvana G. Dellepiane

**Affiliations:** DITEN, Università degli Studi di Genova, Via Opera Pia 11a, 16145 Genova, Italy

## Abstract

This paper describes a method based on an automatic segmentation process to coregister carpal bones of the same patient imaged at different time points. A rigid registration was chosen to avoid artificial bone deformations and to allow finding eventual differences in the bone shape due to erosion, disease regression, or other eventual pathological signs. The actual registration step is performed on the basis of principal inertial axes of each carpal bone volume, as estimated from the inertia matrix. In contrast to already published approaches, the proposed method suggests splitting the 3D rotation into successive rotations about one axis at a time (the so-called basic or elemental rotations). In such a way, singularity and ambiguity drawbacks affecting other classical methods, for instance, the Euler angles method, are addressed. The proposed method was quantitatively evaluated using a set of real magnetic resonance imaging (MRI) sequences acquired at two different times from healthy wrists and by choosing a direct volumetric comparison as a cost function. Both the segmentation and registration steps are not based on a priori models, and they are therefore able to obtain good results even in pathological cases, as proven by the visual evaluation of actual pathological cases.

## 1. Introduction

Diseases related to the wrist, such as rheumatoid arthritis (RA), have a major negative impact on quality of life because they are the most common causes of severe long-term pain and physical disability [1]. An early diagnosis of the pathology in such cases provides the patient with targeted care, a high probability of improvement, and a reduction in social costs [2]. The focus of this work was the erosive arthritis of wrist bones, which is the most disabling rheumatic disease [3].

Even though conventional radiography is considered the traditional gold standard for evaluating bone erosions, poor performances (i.e., overall sensitivity, specificity, and accuracy of 25.5%, 98.3%, and 70.1%, resp.) have been reported for detecting wrist erosions [4]. As clearly stated in [5], computed tomography (CT) has been shown to be more sensitive than radiography but, limited by the radiation dose, it is rarely used in clinical practice. Indeed, MRI allows the simultaneous assessment of synovial membranes, articular fluid, cartilage, bones, ligaments, tendons, and tendon sheaths [6]. MRI can detect erosive RA changes such as bone and cartilage damage with greater sensitivity than conventional radiography [7]. An interesting and clear overview of the clinical problem is given in [5] along with indications for the quantification of RA features from MRI.

For the diagnosis and monitoring of disease progression, a longitudinal study is necessary where the physician conducts several observations of the same subject over a period of time. A major objective is then the definition of a simple but effective procedure for volume registration while preserving the original intensity values. To this end, a simplified method is here proposed whose main objective is the achievement of a limited effect of interpolation-related blurring. The registration result allows the detection of changes in the morphological bone shape, the localization of changes with respect to the bone volume and surface, and the direct grey-level comparison after registration.

Repeatability of the wrist tomographic examination is affected by difficulties in perfectly reproducing the location and orientation of the anatomical district. Even if translation or angular rotation differences are expected to be relatively small, changes more strongly affect the coronal plane than the sagittal and axial planes. With the aim of a direct shape comparison, a 3D registration is then required to align two volumes acquired at different times so that they overlap and a quantitative analysis of changes becomes possible.

In this context, this work proposes a segmentation-based registration method allowing the overlapping of two MRI tomographic spatial sequences so that the shape of a bone at two different instant times can be compared. Such a unimodal 3D-3D multitemporal registration makes it possible to monitor disease progression in the rheumatic wrist. A rigid registration was chosen to avoid artificial bone deformations and to allow finding eventual differences in the bone shape due to erosion, disease regression, or other eventual pathological signs.

The automatic segmentation of the bone of interest from both acquired volumes is the starting point for a good estimate of the location and orientation parameters, which is here performed through the computation of the inertia matrix. The subsequent steps of registration are performed on the basis of principal inertial axes of each volume.

In contrast to more classical approaches, the proposed method suggests splitting the 3D rotation into successive basic rotations about one axis at a time (also termed elemental rotations). In such a way, only a single plane is affected at one time (i.e., coronal, sagittal, and axial plane), starting from the most important one, as indicated by the major principal inertia axis. In addition, singularity and ambiguity drawbacks affecting other classical methods (e.g., the Euler angles method) are addressed.

Depending on the carpal bones, stopping at one basic rotation is sometimes better than performing two rotations or the full 3D transform. In fact, the errors introduced by the interpolation process can be bound to one plane at a time and can be avoided in those planes where the error is larger than the expected registration benefit.

Such a result has been achieved by applying and quantitatively evaluating the proposed method to a set of real tomographic sequences from healthy subjects and by choosing as a cost function a direct volumetric comparison of a wrist bone acquired at two different times. Starting from such empirical results, the suggestion for the best rotation steps for each bone is derived and can be applied also in the pathological situation. The method can also be applied to pathological bones because the segmentation process is based on the tomographic acquisition grey-level values and does not make any use of atlas or anatomical models. Furthermore, bone morphological changes do not affect the registration procedure. The direct application of the method to such pathological cases has been compared with the classical Euler method, showing that singularity and ambiguity drawbacks do not affect the proposed solution.

Due to the good registration result, 3D visualization of the registered bone surfaces could be displayed to assess eventual morphological changes related to pathology evolution, or multiplanar reformatted slice cuts of the two registered volumes could be displayed, along with the original grey levels, to visually compare corresponding slices of a given bone.

Some aspects led to better results for a few bones when applying only one elemental rotation instead of all three (or even the classical rotation matrix):

- Because the first rotation refers to the eigenvector associated with the largest eigenvalue, its effect is the most relevant, it is associated with the most informative content, and it aligns the two bones along their first principal axis.
- The second and the third rotations refer to the second and the third principal axes, which are more critical and might be affected by a larger estimation error.
- Even though repeatability cannot be assured in two different tomographic acquisitions, the flat shape of the hand and wrist assures that differences in the position and orientation likely affect the coronal sections more than the axial or sagittal ones.
- The rotation angle might be so small that the errors introduced by the transformation and interpolation steps are larger than the benefit of the rotation itself.

The paper is structured in the following way. In Section 2, the work is placed in the context of the state-of-the-art methods. In Section 3, the various registration steps and the metrics used for a quantitative evaluation are presented, pointing out that the method is automatic, unsupervised, and not based on models or a priori knowledge, yet it adapts to the image content. In Section 4, the results obtained from the application of the method to MRI volumes of carpal bones are shown, and these findings are discussed in Section 5.

## 2. Previous Works: Image and Volume Registration

The two major registration strategies can be classified into intensity-based and feature-based methods. An intensity-based approach compares pixel/voxel intensity by means of correlation metrics. Feature-based methods find correspondence between significant features such as points, lines, and surfaces, and a geometrical transformation is then determined to map the two volumes [8].

Longitudinal volumetric image analysis is an important topic, especially investigated for brain structure studies. The review paper by Mills and Tamnes [9] gives a comprehensive overview on longitudinal structural imaging for brain development in children and adolescents.

Indeed, from the imaging point of view, brain morphology is very complex and registration turns to be a very crucial task where intensity-based procedures are mainly required. As an example, the research carried out in [10] addresses the analysis of brain atrophy or, more in general, changes in brain size and shape. It is based on an automated linear registration tool, FLIRT (FMRIB's Linear Image Registration Tool) [11], which makes use of the correlation ratio cost function and of a multiscale optimization strategy.

In [12], the quantification of pointwise changes in surface morphology of the bones of the human wrist is addressed based on CT imaging. The proposed method, referred to as Registration-based Bone Morphometry (RBM), consists of two steps: an atlas selection step and an atlas warping step. Statistical group analysis is reported to demonstrate the application of RBM for the comparison of representative carpal bones based on sex and for the comparison of carpal bones of the left and right wrists for individuals. The utility of RBM is also demonstrated in the context of tracking bone erosion status in RA.

The notable brain size and the structured grey-level content assure a reliable statistical information basis for such intensity-based approaches. In contrast, given their unique shape, hand bones are better suited for applications of feature- and geometrical-based methods. In addition, their small size, the MRI low field, and the low spatial resolution make it harder to use intensity-based algorithms which cannot be based on enough statistically significant data.

As previously reported [13], 2D and 3D feature-based image registration approaches can be grouped according to the four basic steps of the registration procedure:* feature detection, feature matching, mapping function design,* and* image transformation and resampling, *which will be recalled in the next paragraphs.

### 2.1. Feature-Based Registration Methods

According to previous reports [13], the use of feature-based methods is recommended if the images contain enough distinctive and easily detectable objects. In medical applications, such a problem is usually faced by interactive selections made by an expert or by introducing extrinsic features, rigidly positioned with respect to the patient (skin markers, screw markers, dental adapters, etc.). Recently, the availability of reliable segmentation results has enabled improvement of such a process: the obtained closed-boundary regions can be used to derive surface or volumetric features, such as in [14], where the cervical spine is segmented and then a 3D articulated registration is applied. In [15], a registration of functional data was performed after the segmentation of the left ventricle.

After the identification of the significant features, their correspondence can be estimated using their descriptors, preferably invariant to the expected image deformation. A group of methods devoted to register satellite images uses moment-based invariants [16] for a description of the segmented regions features. Flusser and Suk used the affine transform invariants [17, 18]; Holm [19] extracted closed-boundary regions and proposed to represent them by perimeter, area, compactness, moments, and moment invariants; and Brivio et al. [20] modeled shadows in mountain images by means of their inertia ellipses. For the stereo-correspondence problem, Bhattacharya and Sinha [21] suggested the application of complex moments.

The mapping function design step, subsequent to the feature correspondence step, is devoted to designing a transform algorithm so that the corresponding features of the two volumes would be as close as possible. At this level, the type of mapping function is chosen, and its parameters are estimated.

Volume registration might be a global or a local process. In the present application, we were interested in coregistration of single bones to monitor the eventual disease evolution. To this end, even though the spatial configuration of the various bones strongly depends on the position of the anatomical part, this problem is solved by using a local registration, where the attention is focused on single bones (volumes of interest) separately. Because each bone is a rigid body, affine transformation is sufficient to achieve a good result and allows for a final morphological comparison.

Finally, the mapping functions constructed at the previous step are applied during the transform step so that the volumes are actually registered.

In the next paragraph, a short overview of the most widely used methods is reported, referring to mapping function design and parametric transformation models addressing the rigid registration. They focus on extraction and correspondence analysis of location and orientation descriptors of the objects of interest and propose a few analytical formulations for the registration solution.

### 2.2. Mapping Functions and Parametric Transformation Models

In space science, the “attitude” of a flying object refers to its orientation with respect to a fixed inertial frame. In robotics, the estimation of orientation and location parameters is a key aspect of rigid-body kinematics. The problem addressed in the present paper has some similarities with these concepts, as the same bone is acquired in a different position and orientation by means of two (nonregistered) tomographic acquisitions performed at some time distance, so that the imaged bone volumes have a different orientation in 3D space.

A very detailed and mathematically sound review of related concepts is given in [22], which was used as a basis for the following considerations.

Let** T** denote an inertial fixed frame whose coordinates are (*x*, *y*, *z*), and let **B** denote the body-attached frame that is free to rotate. Frame **B** is determined by its orthogonal tern (**β**_1_, **β**_2_, **β**_3_)^*T*^.

As defined in [22], the orientation of a rigid body is defined as the orientation of frame **B** with respect to frame **T**. The attitude of a rigid body can be expressed by a variety of mathematical parameterizations, which can be either constrained with redundant elements or unconstrained with minimal elements. The rotation matrix and the unit quaternion are examples of constrained parameterizations. Euler angles, the Rodrigues parameters, and the modified Rodrigues parameters (MRPs) are examples of minimal parameterizations [22].

In practice, the most common orientation representations are the rotation matrix, Euler angles, and unit quaternion. These constitute the basis of most attitude and orientation estimation techniques, and their properties and group structures are of great importance [22].

The Euler angle parameterization is not global, and angles are not well-defined for *θ* = ±*π*/2. This singularity problem is not unique for the Euler angle parameterization but affects all other 3D parameterizations. Unit quaternion is a 4D parameterization, which allows such singularities to be avoided [22].

Both Euler angles and unit quaternions are proven to have a corresponding rotation matrix. Using Euler's theorem, which implies that anybody orientation can be specified in terms of a rotation by some angle about an arbitrary axis, unit quaternions can assume a formulation that gives the corresponding rotation matrix through the Rodrigues formula.

The rotation matrix is a global and unique representation of orientation. By using this matrix, corresponding voxels in coordinates of inertial (**T**) and body (**B**) frames can be mapped to each other as the result of rotations applied to the 3D Euclidean space.

Similar to Euler angles, the rotation matrix can be obtained as a product of three different rotation matrices, each corresponding to an elemental rotation about one of the axes of the fixed coordinate system or about the rotating coordinate system. However, as it was experienced in the present work, for the given application, it is sometimes better to avoid one or even two rotations.

### 2.3. Medical Applications of Moment-Based Registration Methods

In medical applications, registration methods based on principal axes have been applied in different cases of study such as in [23, 24] for MR and positron emission tomography (PET) brain images and in [25] for computed tomography (CT) and PET images. In [26], this method is used for the registration of CT and transmission single-photon emission computed tomography (SPECT) thorax images and for myocardial SPECT stress and rest scans.

Referring to the present application, several papers exist in the literature about the registration of the whole wrist. Most of these studies correlate the registration with the wrist kinematic [27–29], and they consider both the possible rotations of the whole wrist and the movement limits between the singular bones. When focalizing on the wrist bones, such as in [30], a matching between the object of study and a model previously created under different viewing angles is proposed.

In [31], the movements of the wrist are studied, through the registration of the scaphoid bone. In particular, 10 wrist scans are performed from one subject in 10 different positions, and the orientation of the scaphoid, through the determination of the principal axes, is evaluated. The comparison between the different scaphoid bone positions is carried out by registering the volumes with Euler's angles method.

Unlike in [31], our method is not based on the Euler angles and a single rotation matrix, but it performs successive basic rotations to achieve a better registration, as will be demonstrated by our experiments. The relationship between the rotation matrix and the three elemental (i.e., basic) rotation matrices is given in Subsection 1.1 in Supplementary Material available online at https://doi.org/10.1155/2017/7232751.

## 3. The Proposed Approach

The proposed method is not intended to register the whole wrist where kinematic studies are required, and neither does it address the matching with some idealized model or atlas. On the contrary, the purpose of our work is the registration of individual bones, acquired at different times, to detect eventual changes and support the presence of eventual erosion and evolution of the rheumatic disease in general.

The method is supported by a robust, automatic, and reliable segmentation [32, 33] to apply a local registration only to the bone of interest. The volumetric region referring to the bone of interest is extracted independently from the first and second MRI acquisitions.

Based on absolute and central moments of the obtained 3D bone volumes, the proposed method takes the principal axes as feature descriptors and computes them starting from the inertial matrix. The transformation model approach proposes to align both bone volumes to the inertial frame to give them the same location and orientation in 3D space and to allow the subsequent shape comparison. To this purpose, instead of the direct application of one roto-translation matrix, the procedure proposes successive rotations on each 2D plane (along with appropriate translations). The optimization problem is then related to the possible registration parameter configurations, which describe the execution order of the 2D rotation axis (in the maximum of three rotations), the number of such rotations, and the direction of each rotation. Such geometrical considerations, described at Section 3.3, assure that coherent rotations are applied to the two corresponding bone volumes to have them aligned in orientation and in direction as well, without ambiguity.

As a feature-based approach, the estimate of the transformation parameters and the possible configurations are computed in a deterministic way. The cost function adopted is the volumetric comparison between the two aligned bone volumes, as computed by the confusion matrix, which will be described later. Such quantitative metrics have been applied to the available database of real non-pathological cases to drive the selection of the best parameter configuration for each wrist bone type, as explained in the following section.

**Figure 1:**
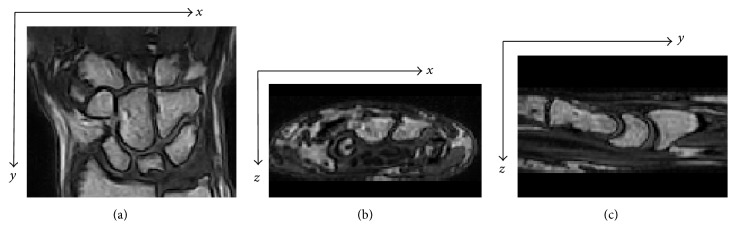
The carpal bones in different visualizations: (a) a coronal section from the original spatial sequence, (b) an axial, and (c) a sagittal section from multiplanar reformatting.

The fixed inertial frame **T** with digital coordinates (*x*, *y*, *z*) is given. Let us start from two MRI spatial sequences, acquired in the coronal plane from the same individual at two instant times *t*_I_ and *t*_II_. From the tomographic sequences, two digital volumes, *V*_I_(*x*, *y*, *z*) and *V*_II_(*x*, *y*, *z*), are generated. Typically, the first (initial) acquisition and the second (follow-up) acquisition are separated by at least a few months in time. These two volumes constitute a mapping whose domains are the voxels coordinate (*x*, *y*, *z*) and codomain is a grey-level value ranging from 0 to 255. For the specific tomograph we used (ESAOTE Artoscan 0.2 Tesla), the image size in the coronal section (represented by [*x*, *y*] coordinates) is 256 × 256 pixels. In the case of anisotropic resolution, an interpolation between coronal slices is performed. The *z* coordinate refers to the normal to the coronal plane and ranges from 0 to *N* − 1, where *N* is the number of coronal slices in the tomographic volume, after the eventual interpolation step. As an example, a coronal slice of a wrist is shown in Figure 1(a), together with an axial and a sagittal section in Figures 1(b) and 1(c), respectively.

### 3.1. Step  1: Feature Detection

A graph-based segmentation [32] is applied to the two tomographic volumes, starting from a seed point that is located by the user inside the bone of interest. The volumes of interest (VOIs) referring to a given carpal bone are thus created, consisting of the binary volumes *b*_I_(*x*, *y*, *z*) and *b*_II_(*x*, *y*, *z*), which represent hereinafter the starting point for the feature descriptor computation.

In other words, the segmentation process (*S*) is a mapping from the original tomographic volume to a binary volume where only voxels belonging to the bone have value 1:

(1) S:VIx,y,z⟶bIx,y,z,S:VIIx,y,z⟶bIIx,y,z,

where

(2) bIx,y,z=1,if  voxelx,y,z  in  VI∈VOII0,otherwise,bIIx,y,z=1,if  voxelx,y,z  in  VII∈VOIII0,otherwise.

### 3.2. Step  2: Feature Description and Matching

**Figure 2:**
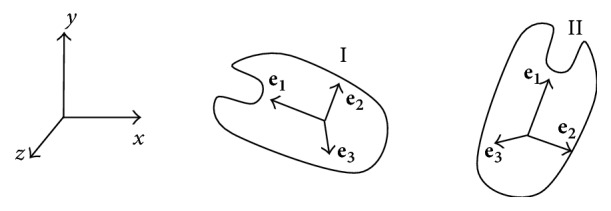
The fixed inertial frame **T**, the first (initial) bone volume (I), and the follow-up bone volume (II), together with their local coordinate frames.

For the two obtained VOIs, the principal axes are extracted as descriptors to be used in the feature correspondence step. They are represented by (**e**_1_, **e**_2_, **e**_3_) terns, as shown in Figure 2, and are the body frame coordinate systems *E*_I_ and *E*_II_, respectively. The detailed algorithm for feature description and matching is described in the following section.

The centroid of both VOIs is computed by applying the first absolute moments of *b*_*W*_(*x*, *y*, *z*), *W* being I or II:

(3) xg=∑x,y,zbWx,y,zx∑x,y,zbWx,y,z,yg=∑x,y,zbWx,y,zy∑x,y,zbWx,y,z,zg=∑x,y,zbWx,y,zz∑x,y,zbWx,y,z.

Such information is needed to perform the translation of the bodies, a necessary phase for the appropriate registration and for the computation of the central moments *M*_*lmn*_ (of order *l*, *m*, *n*) whence the inertia matrix *I*_*W*_ is constructed:

(4) Mlmn=∑x,y,zx−xgl·y−ygm·z−zgn·bWx,y,z,IW=Ixx−Ixy−Ixz−IyxIyy−Iyz−Izx−IzyIzz

having defined

(5) Ixx=M020+M002,Iyy=M200+M002,Izz=M200+M020,Ixy=Iyx=M110,Iyz=Izy=M011,Ixz=Izx=M101.

The matrix of the eigenvectors *E*_*W*_ is finally extracted from the inertia matrix:

(6) $$\begin{array}{c} E_{W}=\left[\begin{array}{ccc} e_{x1} & e_{x2} & e_{x3}\\ e_{y1} & e_{y2} & e_{y3}\\ e_{z1} & e_{z2} & e_{z3} \end{array}\right]. \end{array}$$

The first column is the first principal axis **e**_1_ associated with the largest eigenvalue, the second column is the second principal axis **e**_2_ and the last is the third principal axis **e**_3_.

The purpose of the method is to geometrically register *b*_I_(*x*, *y*, *z*) and *b*_II_(*x*, *y*, *z*) in such a way that their affine transformed versions, *b*_I_^*Ω*^(*x*, *y*, *z*) and *b*_II_^*Ω*^(*x*, *y*, *z*), occupy the same spatial volume and can overlap to evaluate eventual changes. The objective is then the maximization of the intersection volume:

(7) $$\begin{array}{c} b_{X}^{}\left(\;x,y,z\;\right)=b_{I}^{Ω}\left(\;x,y,z\;\right)\cap b_{II}^{Ω}\left(\;x,y,z\;\right). \end{array}$$

At the same time, it is obvious that the correspondence between the descriptors turns to be maximized in the final result if the method is working properly.

The above metrics of (7) can be evaluated by means of the confusion matrix, as shown in Table  1 in Supplementary Material.

Specifically, sensitivity (SENS) and precision (PR) parameters are computed:

(8) SENS=bXx,y,zbIΩx,y,z,PR=bXx,y,zbIIΩx,y,z.

Sensitivity shows eventual loss of bone tissue between the initial and the follow-up acquisition, which might be due to some bone erosion process in pathological situations. Precision indicates a possible presence of voxels in the follow-up bone, which were not depicted in the initial bone, thus indicating, for instance, an eventual reabsorbed edema in pathological cases. In healthy subjects, both indexes should be close to 1, and any deviation from this value indicates imprecisions and processing errors.

Instead of sensitivity versus 1-specificity, the plot of sensitivity versus 1-precision allows a more significant receiver-operating-characteristic (ROC) scatterplot to be drawn and will be used in Results to prove the performances of the proposed method.

### 3.3. Step  3: Transformation Procedure and Parameter Estimation

As already mentioned, the proposed approach is an iterative method that avoids arbitrary rotations in the 3D space but performs sequential basic rotations. At each step, the central inertia matrix of the rigid body is computed together with the eigenvectors. From here, the rotation angle and direction are estimated. After each rotation, the volumes are changed, and then new parameters must be recomputed by means of a new inertia matrix.

From among the possible parametric configurations, the most appropriate one can be evaluated for each carpal bone through application to a dataset of real non-pathological volumes where no changes are expected.

#### 3.3.1. Rotation Angles

After obtaining the eigenvectors of VOI *b*_I_(*x*, *y*, *z*), rotation angles can be estimated. Let us suppose that the VOI is rotated around versor **z** at first, that is, on the coronal plane. The first rotation angle is measured looking at the first eigenvector **e**_1_.

Let the projection of the first principal axis **e**_1_ on the coronal plane (**x**, **y**) represent the following vector, whose components are *e*_*x*1_ and *e*_*y*1_:

(9) $$\begin{array}{c} e_{1}^{xy}=e_{x1}x+e_{y1}y. \end{array}$$

Two possible rotation angles are estimated by looking at the angle formed between vector **e**_1_^*xy*^and versor **x** or the one formed with versor **y**. In the former case, the corresponding angle is

(10) $$\begin{array}{c} \theta_{x}=\cos^{-1}\left(\;\frac{e_{x1}}{\left|x\right|\left|e_{1}^{xy}\right|}\;\right). \end{array}$$

In the latter case, the angle is

(11) $$\begin{array}{c} \theta_{y}=\cos^{-1}\left(\;\frac{e_{y1}}{\left|y\right|\left|e_{1}^{xy}\right|}\;\right). \end{array}$$

The smallest angle between *θ*_*x*_ and *θ*_*y*_ is chosen as the rotation angle *θ*_1_ to be applied for obtaining *b*_I_′(*x*, *y*, *z*). To avoid incongruent alignment, for the follow-up volume *b*_II_(*x*, *y*, *z*), the correspondent angle is chosen and computed. After rotation of both VOIs, a 3D translation of the second volume with respect to the first one is applied. To this purpose, the center of gravity of the rotated bodies is recalculated using (3), and the corresponding translation is carried out so that *b*_II_′(*x*, *y*, *z*) is obtained.

Starting from the newly computed eigenvectors (as described at (4)–(6)), in the next iterative step, the angle value and direction are estimated by repeating the same procedure for a rotation about the **y**-axis, focalizing to the second eigenvector **e**_2_. With the same criterion described above, the new angles *θ*_2_ for VOI_I_ and VOI_II_, respectively, are evaluated. Again, after rotation of both volumes, a 3D translation is applied. This time, *b*_I_′′(*x*, *y*, *z*) and *b*_II_′′(*x*, *y*, *z*) are obtained. The last step also envisioned an equal process, with a rotation in the sagittal plane (around **x**-axis), on the basis of the third eigenvector, followed by a translation. Finally, *b*_I_′′′(*x*, *y*, *z*) and *b*_II_′′′(*x*, *y*, *z*) are obtained.

It can be experimentally demonstrated that the best result is sometimes obtained with less than three rotations. In any case, according to (7), the final results are named *b*_I_^Ω^(*x*, *y*, *z*) and *b*_II_^Ω^(*x*, *y*, *z*).

The proposed method has been implemented in Matlab and the processing time on a i7-7500U, CPU 2.70 GHz, RAM 8 GB computer is approximately 1 minute.

## 4. Results

**Figure 3:**
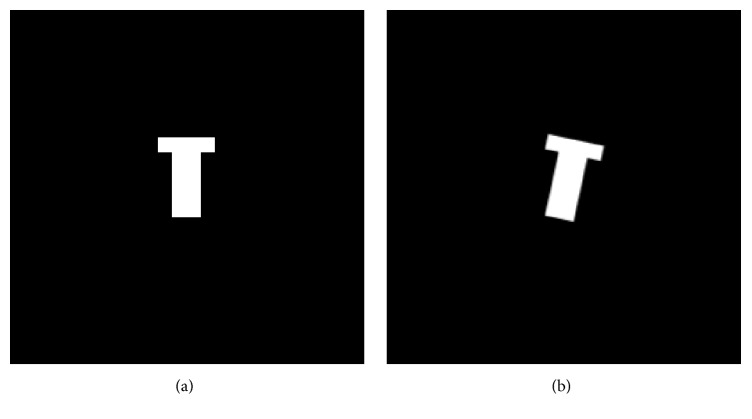
(a) One slice of the synthetic volume and (b) one slice of the rotated synthetic volume.

The proposed registration technique has been tuned and characterized by utilizing a set of synthetic volumes. After a t-shaped volume has been generated, as in Figure 3(a), it was rotated in various ways (see, e.g., Figure 3(b), where one slice is displayed). Considering that a perfect registration is made difficult by the unavoidable interpolation blurring, as well as by the estimation errors, the average sensitivity and precision values of 0.992 and 0.823, respectively, are considered good performances.

In addition, because the applied rotations are known, the error between the computed and actual angles can be evaluated, which is approximately 6 · 10^−3^ radians, in an average.

After evaluation of the synthetic volumes, the method was applied to real cases. These refer to wrist bones acquired in T1-weighted modality using a low-field ESAOTE Artoscan at 0.2 Tesla. All volumes were acquired in the coronal plane, with a resolution of 0.5 × 0.5 mm^2^ and image size of 256 × 256 pixels. Six patients were analyzed, all having an initial and a follow-up tomographic acquisition. Focus of the study was the following eight wrist bones: capitate, scaphoid, lunate, hamate, pisiform, trapezoid, triquetrum, and trapezium. The number of healthy bones on which the method was quantitatively evaluated was 35. A total of 13 pathological bones were also registered, but no quantitative measures were possible in such cases.

**Figure 4:**
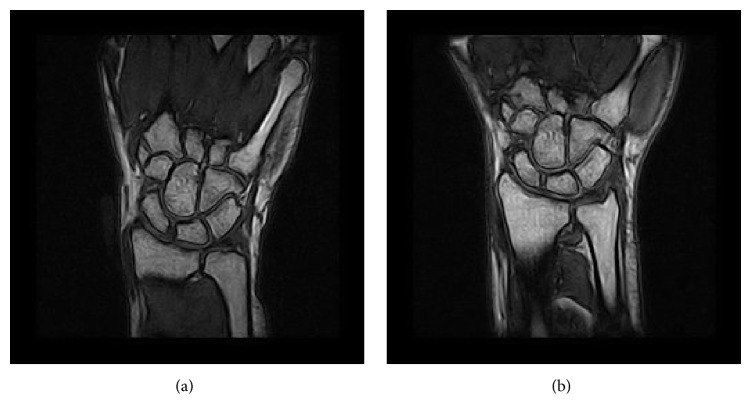
A sample coronal slice from the initial MRI spatial sequence (a) and the follow-up spatial sequence (b).

In Figure 4, it is possible to see the corresponding MRI slices of one patient as acquired at two different times. In particular, in Figure 4(a), the first (initial) volume is shown, and in Figure 4(b), the second (or follow-up) volume is shown.

**Figure 5:**
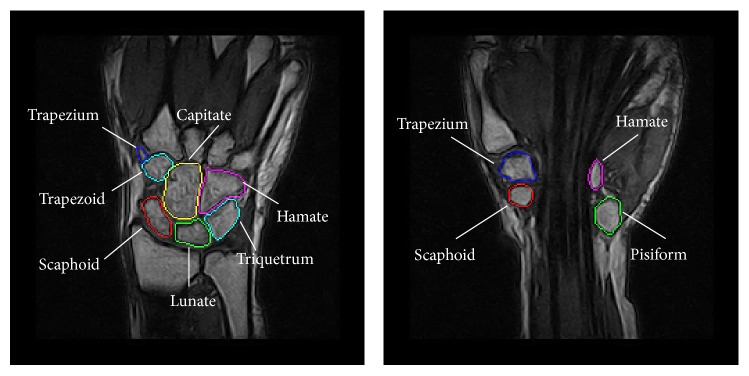
Bone configuration: two sample coronal slices from the first acquisition, along with the contours of the segmented bones.

When required, to make the volume isotropic, an interpolation is applied, thus making the voxel size equal to 0.5 × 0.5 × 0.5 mm^3^. Once the isotropic volumes are obtained, the segmentation is applied with the purpose of extracting individual bones volumes *b*_I_(*x*, *y*, *z*) and *b*_II_(*x*, *y*, *z*) as explained in (2).  Figure 5 shows two sample coronal slices from the first acquisition, along with the contours of the segmented bones.

**Figure 6:**
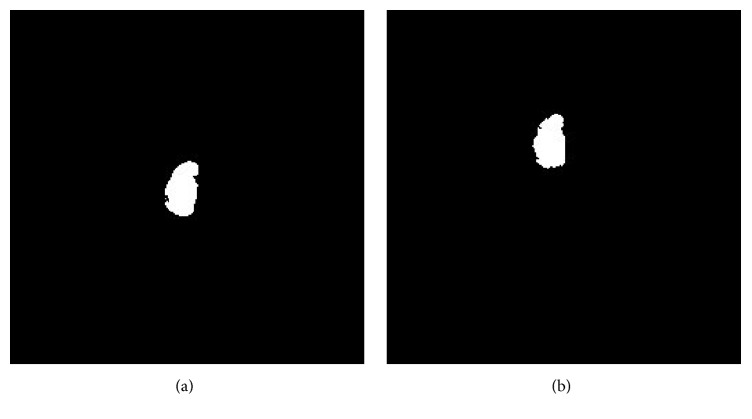
(a) A sample coronal slice of the capitate bone volume segmented from the first acquisition *b*_I_(*x*, *y*, *z*); (b) a sample coronal slice of the capitate bone volume from the follow-up acquisition *b*_II_(*x*, *y*, *z*).

As an example, a coronal section of the capitate bone as segmented from the two original volumes is shown in Figure 6. In addition, according to Figure 4, one can see how the spatial location and orientation of the two bone volumes are very different between the two acquisition times.

**Figure 7:**
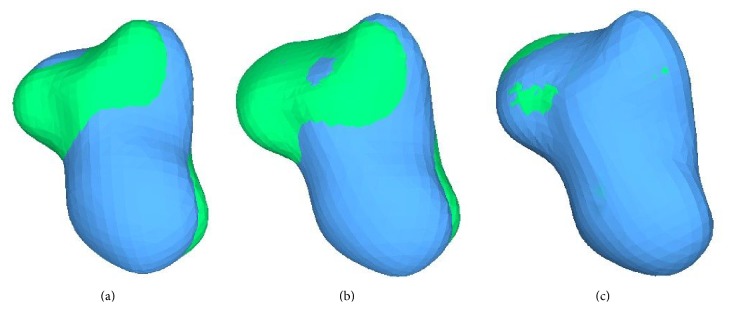
3D surface visualization of the registered capitate bone (initial volume in green, follow-up volume in blue): from (a) first rotation in the coronal plane, (b) second rotation in the axial plane, and (c) third rotation in the sagittal plane.

**Figure 8:**
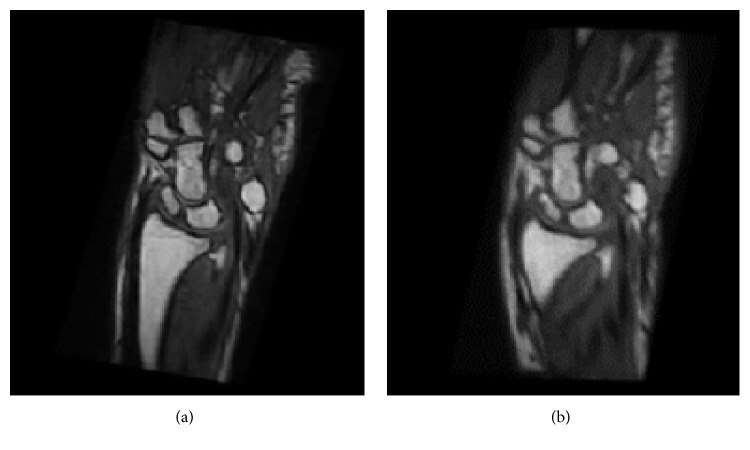
Corresponding sample slices from the first initial MRI sequence (a) and the follow-up sequence (b) after registration of the capitate bone.

For each bone, the proposed registration procedure is applied to volumes *b*_I_(*x*, *y*, *z*) and *b*_II_(*x*, *y*, *z*). In Figure 7, an example of 3D visualization of the three different rotation results as obtained for the capitate bone is shown. The initial bone volume is shown in green, and the follow-up bone is shown in blue. In particular, this figure illustrates the first rotation in the coronal plane (*b*_I_′(*x*, *y*, *z*) and *b*_II_′(*x*, *y*, *z*)), the second rotation in the axial plane (*b*_I_′′(*x*, *y*, *z*) and *b*_II_′′(*x*, *y*, *z*)), and the third rotation in the sagittal plane (*b*_I_′′′(*x*, *y*, *z*) and *b*_II_′′′(*x*, *y*, *z*)).  Figure 8 shows two corresponding sample slices from the first initial MRI sequence and the follow-up sequence after registration of the capitate bone.

The metrics proposed in Section 3.2 allow quantitative evaluation and provide the possibility of assessing, for each bone, the opportunity to carry out all three rotations or less.

**Figure 9:**
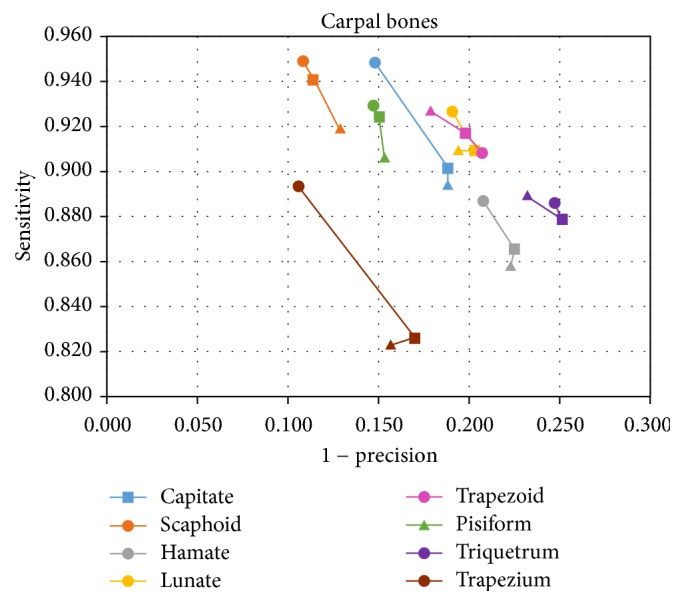
ROC plot, where triangles correspond to one rotation, squares to two rotations, and circles to three rotations.

Figure 9 reports the results of the proposed registration as obtained with healthy bones, which do not show signs of erosion or edema. In the abscissa, there is the parameter 1-precision, while, in the ordinate, there is the parameter sensitivity, as defined in (8). The ideal case of perfect registration corresponds to values near 0 for the former parameter and values close to 1 for the latter parameter.

For each bone type, a triangle corresponds to the performance of the first basic rotation, a square to two rotations, and a circle to three rotations. As one can notice, all of the bones, except the trapezoid and triquetrum, obtained the best registration result with three rotations. For the other two bones, one rotation was sufficient.

The trapezium showed the most remarkable improvement when comparing the first, second, and third rotations. The same trend, although a little less considerable, characterized the hamate and capitate bones. For this last bone, the result of the quantitative assessment was in line with the visual evaluation. In fact, as shown in Figure 7, the volumes obtained with the third rotation showed a better registration than those obtained with the other rotations.

In general, the obtained results were satisfactory, with all showing sensitivity values larger than 0.89 and precision values larger than 0.77 (the worst result being reported for the triquetrum). The best registration result was obtained for the scaphoid, with a sensitivity value of 0.95 and precision value of 0.89.

As an argumentation with respect to the more classical Euler's angles method, the same healthy cases were registered by applying the single classical rotation matrix (Direction Cosine Matrix), and a subsequent quantitative evaluation was carried out. Additionally, in this case, the Euler angles were estimated from the analysis of the principal inertia axes of the segmented bone volumes. As an example of a quantitative comparison, Table 1 shows the sensitivity and precision values as averaged over all of the available non-pathological scaphoid bones.

The advantage of the proposed method is due to two main reasons. In fact, the Euler method suffers from well-known problems such as orientation ambiguity and non-singularity, which do not affect the proposed approach. In addition, with the proposed method, the best result could be obtained after evaluating one, two, or three rotations based on the specific bone (indeed, in the scaphoid case, three rotations were considered the best solution, as shown in Figure 9).

The lower values for the sensitivity and precision of the Euler method are actually due to the fact that, for some scaphoid cases, the two volumes have been registered with a correct direction but a wrong orientation of one axis. In other words, the bottom and top of the two bones are not correctly overlapping.

After confirming that the registration results were satisfactory and accurate when dealing with non-pathological bones, the method was applied to pathological cases to verify its applicability for the assessment of disease evolution. As mentioned previously, rheumatologists affirmed the presence of rheumatoid arthritis on 13 different bones in the available database. The pathological signs only locally affected bone morphology, and the preserved similarity between the initial and follow-up bone volumes enabled a sufficiently good registration. In addition, the proposed segmentation method, which does not make use of a priori knowledge or an anatomical model, was able to extract in a correct way the bone volumes, despite erosion or other pathological signs.

**Figure 10:**
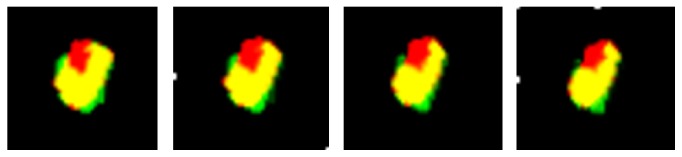
A pathological trapezoid bone: fusion of initial and follow-up volumes.

As an example, a pathological trapezoid bone is shown in Figure 10 after registration. Four slices of the registered volumes are reported where yellow pixels indicate a perfect overlap between initial and follow-up volumes. The green color is associated with voxels that belong to the follow-up volume but not to the initial volume, while voxels belonging to the initial volume but not to the follow-up volume are those colored in red.

As a consequence, red pixels correspond to the erosion process, while green pixels indicate more probable errors of the registration processing chain (i.e., actual registration errors along with segmentation errors). Even in this given case, where significant erosion was shown to affect the bone, the registration result was very accurate, proving that the pathological sign does not invalidate the proposed method.

## 5. Discussions and Conclusion

The proposed method, starting from intra-patient tomographic volumes acquired at different time points, proved to reliably coregister carpal bones, in order to detect eventual morphological changes. The approach operates directly on MRI spatial sequences, without any atlas or anatomical model and was also shown to be applicable to pathological cases. The retrospective analysis of given cases represents a feasibility study proving the effectiveness of the proposed method for tracking the progression of erosion or edema related to rheumatoid arthritis.

In contrast to other 3D model registration approaches, which are based on surface feature representations and matching, the present method is volume-based. Compared with the more classical Euler angles, it does not suffer from problems such as the ambiguity in axis direction and the fact that an infinite number of rotations cannot be parametrized. In addition, it is able to reduce the interpolation blurring effect.

The assessment of the method's performance was based on healthy bones where no significant changes were expected from the initial to the follow-up study, and a quantitative evaluation was carried out, thus objectively demonstrating the correctness and validity of the method. In these cases, the registration purpose is a perfect result, where deviations are only due to errors or approximations associated with the phases of parameter estimation and interpolation, in addition to eventual segmentation errors. For the specific registration procedure, no phantom or other ground-truth standard data are required because the direct comparison of corresponding bones that are not changed over time is possible.

Indeed, the proposed method was also applied to pathological studies, the location and size of the pathology being identified by specialist physicians. Bone erosion only locally affected the bone volume of interest, and changes were minimal and limited, which showed that the method can produce satisfactory results in pathological cases as well. This approach allows tracking changes in bone morphology without these changes affecting the registration process.

When comparing with published literature, quantitative evaluation of individual cases is very seldom available, especially when dealing with longitudinal wrist bone analysis. In some cases, such as in [5], shape changes are manually evaluated.

In the statistical study presented in [12], the average RMS surface distance between registered bone and atlas is reported to be 0.25 mm. Starting from the accuracy measures reported at Figure 9, an average distance between the first and the second bone is estimated to be around 0.27 mm in the present study. By taking into account the fact that the CT voxel resolution was 0.32 × 0.32 × 0.26 (mm^3^) in that study, while it is 0.5 × 0.5 × 0.5 (mm^3^) in the current one, the present performances compare favorably.

In conclusion, the present feasibility study, starting from low-field MRI spatial sequences, proved that it is possible to automatically quantify bone erosion by simple image processing algorithms, without a priori knowledge or models. Innovative aspects rely on the fact that the proposed method is an automatic, fast, and simple image processing procedure for quantitative analysis in longitudinal wrist studies. Being based on the only original MRI grey levels, no use of a priori knowledge or models is required.

After this preliminary study, a rigorous clinical validation of the method in pathological cases is planned as a future activity.

## Supplementary Material

Subsection *1.1 (3D rotation and basic rotations)* shows how the rotation matrix can be obtained as a product of three different rotation matrices, each corresponding to an elemental (i.e., basic) rotation about one of the axes of the fixed coordinate system or about the rotating coordinate system.
Subsection *1.2* gives the definition of the Confusion Matrix along with Sensitivity (SENS) and Precision (PR).

## Figures and Tables

**Table 1:** Evaluation of Euler's method and our method.

Scaphoid	Sensitivity	Precision
Euler's method	0.737	0.672
Our method	0.949	0.891
