# Supplementary material for: Multitemporal Volume Registration for the Analysis of Rheumatoid Arthritis Evolution in the Wrist
Source: Int J Biomed Imaging. 2017 Oct 19;2017:7232751. doi: 10.1155/2017/7232751 (PMC5672126; doi:10.1155/2017/7232751)
Supplement: Supplementary file 1 — Subsection 1.1 (3D rotation and basic rotations) shows how the rotation matrix can be obtained as a product of three different rotation matrices, each corresponding to an elemental (i.e., basic) rotation about one of the axes of the fixed coordinate system or about the rotating coordinate system. Subsection 1.2 gives the definition of the Confusion Matrix along with Sensitivity (SENS) and Precision (PR). [file 7232751.f1.docx]

**Supplement to**

**Multi-temporal volume registration**

**for the analysis of rheumatoid arthritis evolution in the wrist**

Roberta Ferretti, Silvana G. Dellepiane*

Università degli Studi di Genova, DITEN, via Opera pia 11a 16145, Genova, Italy

roberta.ferretti@edu.unige.it, silvana.dellepiane@unige.it

- 1. *3D rotation and basic rotations*

The Direction Cosine Matrix (DCM), commonly known as the rotation matrix, R, is the most straightforward implementation of affine geometrical transformation.

Its definition:

$R∊\mathbb{R}^{3\times3}\mid R^{T}R=R R^{T},\det\left( R \right)=1$ (1)

$R=\left[ \begin{matrix} \beta_{x1} & \beta_{x2} & \beta_{x3} \\ {\beta_{y1}} & \beta_{y2} & \beta_{y3} \\ {\beta_{z1}} & {\beta_{z2}} & \beta_{z3} \end{matrix} \right]$ (2)

points to the orthogonal basis of columns, making it an orthogonal matrix.

The rotation matrix is a global and unique representation of orientation. By using this matrix, corresponding voxels in coordinates of inertial (**T**) and body (**B**) frames can be mapped to each other. The registration is then the result of rotations applied to all 3D Euclidean space.

The Euler angles describe the orientation of a rigid body in 3D space. Because any orientation can be achieved by composing three elemental rotations, three parametric angles are needed, which can be given in several ways. The specific sequence of rotation axes gives rise to one of the twelve different configurations. We are here referring to the z-x-y configuration.

Given any two coordinate systems, the process by which the three Euler angles transform the first coordinate system into the second system can be summarized in the following procedure, where the line of nodes is an intermediate axis:

1. A positive rotation by an angle ψ about one axis of the first system (z-axis);

2. A positive rotation by an angle θ about the line of nodes;

3. A positive rotation by an angle φ about one axis of the second system (y-axis).

Similarly, the rotation matrix can be obtained as a product of three different rotation matrices, each corresponding to an elemental rotation about one of the axes of the fixed coordinate system or about the rotating coordinate system:

$R=\left[ \begin{matrix} c\psi& -s\psi& 0 \\ s\psi& c\psi& 0 \\ 0 & 0 & 1 \end{matrix} \right]\left[ \begin{matrix} c\theta& 0 & s\theta\\ 0 & 1 & 0 \\ -s\theta& 0 & c\theta\end{matrix} \right]\left[ \begin{matrix} 1 & 0 & 0 \\ 0 & c\varphi& -s\varphi\\ 0 & s\varphi& c\varphi\end{matrix} \right]$ (3)

where *c* and *s* refer to cosine and sine, respectively.

Each of the three matrices in equation (3) refers to a basic rotation because one axis is fixed. The registration transformation (along with the interpolation) can be performed layer by layer, thus combining only voxels that belong to one of the tomographic planes at a time.

- 1. *Confusion matrix*

The confusion matrix related to the present study is shown in Table 1, where $b_{I}^{\Omega}$and $b_{II}^{\Omega}$ refer to voxels belonging to the rotated first and second bone volumes, respectively. $\bar{b_{I}^{\Omega}}$and $\bar{b_{II}^{\Omega}}$ refer to voxels not belonging to the two bone volumes, respectively.

Table 1: Confusion matrix

|  | $b_{I}^{\Omega}$ | $\bar{b_{I}^{\Omega}}$ |
| --- | --- | --- |
| $b_{II}^{\Omega}$ | *N_00_* | *N_01_* |
| $\bar{b_{II}^{\Omega}}$ | *N_10_* | *N_11_* |

As a consequence, *N_00_* indicates the number of voxels belonging to both volumes and is in fact the cardinality of $b_{X}\left( x,y,z \right)$ (i.e.,$\left| b_{X}\left( x,y,z \right) \right|$) which was defined in paper equation (7). *N_10_* should correspond to voxels belonging to the Initial but not to the Follow-up bone, and vice versa for *N_01_. N_11_* indicates the number of voxels that do not belong to one or the other VOI. This last value is much larger than the other table elements and might have an arbitrary size, depending on the number of considered slices in the original volumes.

From among the important parameters that can be computed from the confusion matrix, Sensitivity (SENS) and Precision (PR) are here taken into account according to the following formula:

$SENS=\frac{N_{00}}{N_{00}+N_{10}} PR=\frac{N_{00}}{N_{00}+N_{01}}$,

Despite many papers take into account Specificity parameter instead of Precision, it was here decided to avoid its use. In fact, since Specificity is computed on the basis of *N_11_*_,_ its value is always very close to 1 and is not significant in the present application where the volumes of interest are very small with respect to the background.

Instead of Sensitivity versus 1-Specificity, the plot of Sensitivity versus 1-Precision allows a more significant receiver-operating-characteristic (ROC) scatterplot to be drawn.
